# Enhancing physiology learning through group dynamics: outcomes and perceptions of medical students

**DOI:** 10.3389/fphys.2025.1662624

**Published:** 2025-10-21

**Authors:** Antonio S. Tutor, Maria Del Nogal Avila, Úrsula Muñoz, Isabel Sánchez-Vera

**Affiliations:** Departamento de Ciencias Médicas Básicas, Facultad de Medicina, Universidad San Pablo-CEU, CEU Universities, Madrid, Spain

**Keywords:** active learning, decision-making, teamwork, group dynamics, higher education, physiology education

## Abstract

**Background:**

In health sciences education, active learning strategies are increasingly recognized for their role in enhancing student engagement and competency development. This study explores the impact of a structured group dynamics activity, framed within a desert survival scenario, on decision-making, teamwork, and academic performance among second-year medical students studying renal physiology.

**Methods:**

A mixed-methods design was used involving 195 students, 140 of whom participated in a two-session collaborative activity grounded in physiological knowledge. The intervention emphasized individual and collective decision-making, followed by a teamwork-based quiz to reinforce theoretical content. Academic performance was evaluated using scores from a standardized multiple-choice exam. Student perceptions were gathered through Likert-scale and open-ended surveys.

**Results:**

Students who participated in the group activity showed significantly higher percentages of correct answers in the renal physiology exam section compared to non-participants (p < 0.05), suggesting a positive association with improved performance and fewer incorrect or unanswered items. Differences in other content blocks (digestive and endocrine physiology) were smaller and not statistically significant. Survey responses indicated overwhelmingly positive perceptions of the activity, particularly regarding its role in reinforcing knowledge, promoting collaborative skills, and fostering an engaging learning environment.

**Conclusion:**

These results suggest that participation in a group dynamic activity during physiology sessions could be associated with better academic results related to the content of this activity. While this exploratory study cannot establish causal relationships based on its design, the activity appears particularly beneficial when the scenario design aligns with course content. These results support the pedagogical value of active and collaborative learning in medical education, encouraging the implementation of similar interventions. However, future research should focus on conducting randomized controlled trials with long-term follow-up to establish causality and retention effects.

## 1 Introduction

Continual advancements in healthcare practices and the escalating complexity of clinical environments require a systematic and ongoing reevaluation of pedagogical approaches in health sciences education (Gonzalo et al., 2022; Tutor et al., 2023). Traditional didactic methods have historically been central to disseminating foundational theoretical knowledge. However, they are now considered inadequate for equipping students with the competencies necessary for navigating the multifaceted challenges of contemporary clinical practice (Hora, 2019; McGunagle and Zizka, 2020). Consequently, active learning paradigms have gained prominence due to their ability to promote student engagement, critical thinking, and the practical application of knowledge—essential attributes for becoming proficient healthcare professionals (Michael, 2006; Harden et al., 2024).

In this context, collaborative learning methodologies, particularly those based on group dynamics principles, have been shown to significantly increase academic achievement and develop essential professional skills (Marasri, 2025; Zaafour et al., 2025). Participating in group activities not only consolidates theoretical knowledge, but also develops interpersonal and cognitive skills, such as communication, teamwork, problem-solving, and collective decision-making (James et al., 2022). Vygotsky’s social constructivist framework, particularly the Zone of Proximal Development (ZPD), provides the theoretical foundation for understanding these outcomes, as students reach higher levels of comprehension through guided peer interaction than they could achieve individually (Sanders and Welk, 2005; Kantar et al., 2020). In the desert survival simulation, collaboration enables learners to co-construct knowledge, negotiate solutions, and extend their reasoning beyond individual capacity.

Complementary cognitive perspectives further strengthen this rationale. Cognitive Load Theory suggests that contextualized learning environments, such as survival-based scenarios, reduce extraneous cognitive demands and allow students to concentrate on processing complex physiological concepts (Young et al., 2014; Szulewski et al., 2021). Kolb’s Experiential Learning Theory explains how the desert survival activity aligns with the cyclical process of concrete experience, reflective observation, abstract conceptualization, and active experimentation. In this context, students first engage in the survival scenario (concrete experience), then reflect on their decisions (reflective observation), connect these reflections with physiological principles (abstract conceptualization), and finally apply their insights in subsequent tasks (active experimentation) (Wijnen-Meijer et al., 2022). Elaboration Theory additionally supports the hypothesis that linking survival decisions to underlying physiological mechanisms promotes meaningful associations, enhancing deep cognitive processing and long-term retention (Van Blankenstein et al., 2013; Haji et al., 2015).

Importantly, the desert survival scenario is uniquely suited for teaching renal physiology because the scenario naturally revolves around water scarcity, fluid balance, and prioritization of limited resources. These elements directly mirror the core functions of the renal system in maintaining homeostasis, thus providing a pedagogically and theoretically justified context that strengthens both comprehension and application of physiological knowledge.

These competencies are critical in modern healthcare settings, which are characterized by interdisciplinary collaboration and integrative patient care.

The contemporary healthcare environment demands practitioners who can function within dynamic teams, manage high-pressure scenarios, and synthesize diverse perspectives to deliver holistic, patient-centered interventions (Sánchez et al., 2024). Pedagogical strategies involving case-based discussions, collaborative problem-solving exercises, and simulation-based training provide students with invaluable opportunities to apply theoretical knowledge in authentic, practice-oriented contexts (Agostino et al., 2024; Alenazi et al., 2024; Agostino et al., 2025). Furthermore, these approaches encourage metacognitive reflection, enabling learners to critically evaluate their cognitive processes and identify areas for improvement. These methodologies also contribute to the development of interpersonal skills that are essential for effective communication and conflict resolution in team-based clinical settings (Frank et al., 2010; Pires et al., 2025).

Existing literature supports the positive impact of collaborative learning on variables such as student motivation, depth of comprehension, knowledge retention, and the ability to apply theoretical constructs to clinical scenarios (Vázquez-García, 2018; Ghani et al., 2021; Zhang and Ma, 2023; Alexander et al., 2024). Empirical evidence consistently shows that learners in group-based educational settings have a better understanding of concepts and are better able to apply them (Michaelsen et al., 2023). Additionally, participation in such collaborative settings fosters motivational enhancement, interpersonal skill development, and the cultivation of a supportive educational environment. Despite the widespread acknowledgment of these cognitive and socioemotional benefits, further empirical research is necessary to understand the direct impact of these instructional strategies on academic performance in health sciences curricula.

Despite the documented advantages, the integration of active learning approaches in health sciences education faces significant resistance from faculty and students alike (Hawkins et al., 2015; Harden and Laidlaw, 2020; Børt et al., 2023). Concerns include potential deficits in content coverage, inequitable contributions among group members, and entrenched preferences for traditional, individualized learning models. Effectively managing group dynamics, including equitable participation, conflict mediation, and accommodating diverse learner proficiencies, constitutes a significant pedagogical challenge. This resistance is exacerbated by the institutional inertia of lecture-centric pedagogies, highlighting the difficulty of shifting toward learner-centered instructional frameworks.

The present study examines these challenges by investigating health sciences students’ perceptions after participating in a structured, group-based learning intervention. The study aims to elucidate the extent to which collaborative activities reinforce theoretical knowledge, foster the development of teamwork competencies, and facilitate the application of theoretical constructs in practical scenarios. Additionally, the study compares academic outcomes between cohorts engaged in collaborative versus traditional, individualistic learning modalities.

This study aims to explore the effectiveness of group-based teaching methods in improving academic performance and professional skills development. The primary outcome is performance in the renal physiology exam. The secondary outcomes include the development of collaborative skills, perceptions of the learning experience, and the practical application of theoretical knowledge in clinical settings. Ultimately, the results will contribute to expanding knowledge about active learning through group dynamics to transform health sciences education and better prepare future professionals for the collaborative nature of modern clinical practice.

## 2 Materials and methods

This research was carried out in the context of the subject Physiology II, a compulsory course during the second academic year of Medical Degree at our university. This subject of 9 ECTS credits involves the in-depth study of the physiological intricacies of the digestive, renal, and endocrine systems. The importance of this subject lies in the fact that it allows students to understand the physiology of the different systems and to discern the influence of the failure of some pathways in the development of numerous diseases that will be taught in more advanced courses.

Students have been invited to participate in an activity that will consist of two sessions of 2 hours each, based on group dynamics to learn, understand, and to know how to apply the knowledge about the function of the renal system and adaptation to the environment. This activity was developed after completing the theoretical classes that cover the subject related to renal physiology. As attendance was voluntary, participation in this activity did not count towards students’ final grade for the course. Students were simply encouraged to participate in order to review the concepts of renal physiology.

Groups were limited to 5–6 students to balance participation opportunities and maintain manageable interaction, following recommendations from cooperative learning literature (Slavin, 1995; Johnson et al., 1998). Each session was set at 2 hours to allow sufficient time for all phases of decision-making and teamwork without inducing fatigue, as suggested in prior studies on active learning.

### 2.1 Group dynamics methodology

Students were confronted with a simulated desert survival scenario inspired by previously validated group dynamics frameworks (Lafferty et al., 1974). The starting situation was to assume that they were lost in the desert after a trip to Marrakech. The environmental and situational parameters were carefully outlined to immerse the participants in the scenario.

**FIGURE 1:**
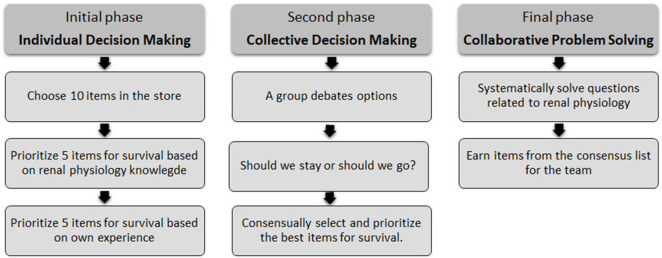
Stages of the group dynamics activity in the desert survival scenario. Schematic representation of the three sequential phases of the group dynamics activity: (1) individual decision-making, (2) consensus-based group decision-making, and (3) collaborative problem-solving applied to renal physiology content.

Group dynamics were developed in three distinct stages (Figure 1). The first and second phases focused primarily on decision-making, based on previous knowledge in Physiology. The first stage required students to make individual decisions and in the second stage they had to consensualize those decisions within the group members. Progression to the third phase depended on achieving a unified decision.

The overall goal of the third phase was to enhance teamwork by reviewing the content of renal physiology. Participants had to answer a series of questions and challenges related to the topic. For each correct answer the group earned an item, with a pre-set score determined by the instructors. Importantly, the participants were unaware of the scoring details during the activity. The accumulated scores determined the winning group, highlighting their skill in both answering the questions and strategically selecting objects to survive in the wilderness. The selection group consisted of 29 items (Supplementary Table S1), each associated with potential survival benefits. These included toiletries, food and drink, literature on desert flora and fauna, and other tools and supplies. Students were asked to make strategic choices based on their perceived utility in a desert survival context. The scoring system was designed by instructors through an initial individual assessment of survival relevance and physiological reasoning, followed by group discussion and consensus to establish the final scoring system.

The instructions explicitly communicated that the group’s survival depended on collective decision-making. It was emphasized that the unity of the group was a critical factor influencing its chances of survival. The various challenges proposed emphasized thoughtful decision-making based on the knowledge previously acquired in the renal physiology classes, as opposed to speed of decision-making. Participants were encouraged to foster a respectful environment, collaborate effectively, and learn from each other, emphasizing the equal importance of each member to the survival of the group.

#### 2.1.1 First phase: individual decision-making

The first phase of the activity was designed to work on decision-making skills, taking advantage of previous theoretical knowledge of Physiology. Students were asked to decide individually on two basic questions: 1) Which out of ten items from the aforementioned list of twenty-nine would they purchase in a store before starting their trip; 2) From the ten items chosen, they were asked to select and rank in order of priority the five most suitable items, based on their knowledge of the body fluids homeostasis, acquired in previous classes on renal physiology, to facilitate survival in a desert environment, and then again select and rank the five most optimal items for survival in the desert, based now on knowledge acquired outside of academia.

#### 2.1.2 Second phase: collective decision-making

All students were randomly divided into 24 groups of five or six, with each quintet or sextet representing a group of friends lost in the desert. This phase of the group dynamics focused on reaching a consensus decision within the group. Participants had to debate and decide whether to stay in the vehicle until rescue services arrived or to venture out into the desert in search of help. In addition, the group had to come up with a unique list of the ten optimal items for survival in the desert from all the items they had individually selected in the previous phase. It was imperative to reach a unanimous group decision, which required the agreement of all members. Although not all members had to fully agree with the final list, the entire group had to agree with the agreed-upon decision. Proceeding to the third phase depended on the formulation of a unified group decision.

#### 2.1.3 Third phase: collaborative problem solving (teamwork)

The final phase consisted of systematically solving the proposed questions and/or problems related to the physiology of the renal system. Each time a team consistently answered one of the challenges, its answer was reviewed by the instructors. A correct answer to a question gave the group the privilege of acquiring an object from the predetermined list, in the order previously determined by the group. At the end of the allotted time, the instructors collected the items earned by each team and then assigned a final overall score based on the group’s cumulative performance.

### 2.2 Data collection and statistical analysis

The study measured the time students took to make decisions both individually and in groups and examined whether there was a association between decision time and the success of object selection outcomes. The percentage of agreement between individual lists, group lists, and an optimal list predefined by the instructors was also calculated. In addition, data were collected on the percentage of correct, incorrect, and unanswered questions in the final exam covering renal, digestive, and endocrine physiology.

Statistical analysis was performed using the SPSS Statistics for Windows, Version 27.0 (Armonk, NY: IBM Corp.), with a significance level set at α = 0.05. P-values below this threshold were considered indicative of statistically significant differences between the groups. The results were expressed as the mean ± standard deviation. The normality of the distributions was assessed using the Shapiro-Wilk test and homogeneity of variances via Levene’s test. The results were compared between two groups: students who participated in the group dynamics activity and those who did not. A Mann–Whitney test was performed to compare the distributions of these two groups and determine whether there were any statistically significant differences, given that the distribution of the non-participants group was not normal. Because group identifiers were not recorded, students were treated as independent observations for all analyses. This assumption is acknowledged as a limitation and is further discussed later in the manuscript. The p-value was corrected to reduce the possible type I error by multiplying it by the number of blocks. The associations between variables were calculated using Spearman’s correlation coefficient. For the effect sizes Cohen’s conventions has been used, whereby d equals 0.2 for a small effect, 0.5 for a medium effect and 0.8 for a large effect.

### 2.3 Students’ perception

The evaluation of students’ perspectives on decision-making and teamwork was conducted through an opinion survey. Although no formal validation process was carried out, the survey was reviewed for clarity by three physiology teachers, and a small group of students who were not involved in the main study, to ensure the comprehensibility and appropriate interpretation of the items. The survey encompassed 11 questions where students expressed their agreement, a question addressing the overall opinion of the group dynamics and an open-ended query (Supplementary Table S2). The survey was distributed to all participants in the group dynamics activity. Participation was voluntary and anonymous, with informed consent obtained from all respondents. We have grouped the answers of the survey questions into three descriptive categories used solely to organize and present the data more clearly: (1) decision-making and changes of opinion, (2) perceptions of teamwork and (3) an overall evaluation of the group dynamics. For a better understanding of the students’ responses in the open-ended query included in Supplementary Table S2, a descriptive thematic analysis was conducted. Two authors independently reviewed and coded the responses, reaching consensus through discussion. Based on this process, five different themes were identified: those related to teamwork and socialization, knowledge reinforcement, practical application of learning, dynamic and fun learning environment, and overall positive perception.

This study was approved by the Ethics Committee of CEU San Pablo University under protocol number 695/23/69. An informed consent was obtained from all students who wished to participate in the survey. Participation was voluntary and participants were assured of confidentiality and unrelated to course grading, with assurances provided to minimize potential perceptions of coercion.

## 3 Results

### 3.1 Profile of participants

The study involved 140 students who participated in the activity, making up 71.8% of the enrolled students for the 2022–2023 academic year. There were also 55 students who chose not to participate, representing 28.2% of the total. Among the participants, the average age was 19 years, ranging from 18 to 25 years. Of these, 29 were male (20.7%) and 111 were female (79.3%).

### 3.2 Initial phase: individual decision-making

Students were asked to individually decide which items to buy in a store. Subsequently, they had to select the optimal five items for survival in a desert based on their prior knowledge of physiology and another set of five items based on their general knowledge of daily life.

**FIGURE 2:**
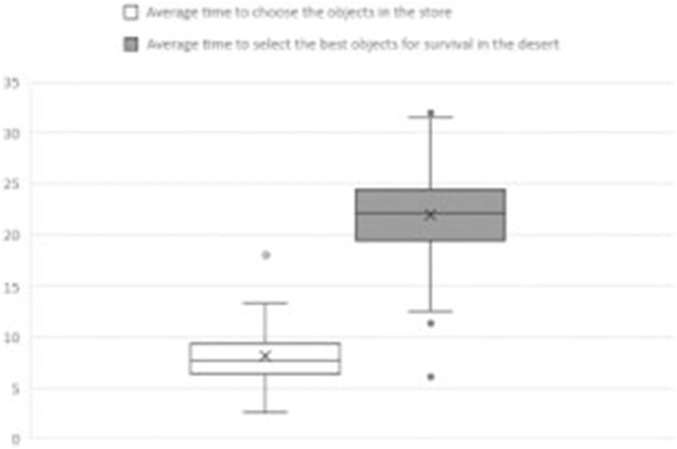
Average time spent by students during the individual object selection process. Mean time (±SD) students spent choosing objects to purchase and ranking the five most suitable items for desert survival based on physiological knowledge.

The mean duration of the initial individual decision-making process (what to purchase in the store) was 8.1 ± 2.3 min, with a range from 2.9 to 18 min. The mean duration of the second individual decision-making process (determining which items were more optimal) was 22 ± 4.4 min, with a range from 6.18 to 32 min (see Figure 2). These results indicate that selecting the top ten items takes longer, suggesting that a foundation of physiological knowledge related to body fluids homeostasis is necessary to make more informed choices.

To assess whether the amount of time students spent making decisions influenced the accuracy of their choices, we examined the degree of agreement between the objects selected and those predetermined by the instructors (see Supplementary Table S1) as optimal choices for survival in the desert based on physiological considerations (Table 1) and based on one’s own experience (Table 2). No significant relationship was found between the time taken to decide and the quality of the decision. Those who spent more time did not necessarily make better choices (r = −0.057; p = 0.505).

**TABLE 1:** Agreement between individual object selection and the optimal list based on physiological criteria. Distribution of the number of correctly selected objects, number and percentage of students selecting them, average decision time, and time range.

Number of correct chosen objects	Number of students who had chosen the correct objects	Students’ percentage (%)	Average time spent choosing the objects (minutes ±SD)	Range (minimum-maximum) (minutes)	95% CI (lower-upper)
1	30	21.4	23.5 ± 3.9	17.25–32	21.99–24.93
2	47	33.6	21.1 ± 4.3	11.33–28.40	19.81–22.35
3	46	32.9	22.1 ± 4. 1	13.51–30.52	20.89–23.34
4	12	8.6	22 ± 6.3	6.11–30.35	18.01–26.06
5	5	3.6	20.8 ± 5.1	15.46–27.20	14.43–27.09

**TABLE 2:** Agreement between individual object selection and the optimal list based on personal experience. Results showing the number of correct objects selected based on general knowledge, including the number and percentage of students, mean decision time, and time range.

Number of correct chosen objects	Number of students who had chosen the correct objects	Students’ percentage (%)	Average time spent choosing the objects (minutes± SD)	Range (mínimum-maximum) (minutes)	95% CI (lower-upper)
0	10	7.1	18.9 ± 4	12.52–23.10	16.08–21.77
1	25	17.9	22.7 ± 3.1	17.5–30.35	21.47–23.99
2	37	26.4	21.8 ± 4.6	13.4–30.52	20.23–23.28
3	44	31.4	22.2 ± 4.9	6.11–31.22	20.74–23.72
4	20	14.3	22.9 ± 4.6	11.33–32	20.76–25.11
5	4	2.9	20.2 ± 3	16.25–22.7	15.51–24.97

Furthermore, we aimed to investigate whether gender had any impact on the duration of decision-making. Our analysis revealed no statistically significant differences in the time spent on decision-making between males (22.5 ± 4.7 min) and females (21.9 ± 4.4 min) (p = 0.796).

### 3.3 Second phase: collective decision-making

Regarding the decision based on prior knowledge in physiology, for which each group had to reach a consensus, 21 groups (87.5%) decided to stay and wait, while three groups decided to go and seek help (12.5%). Therefore, we can affirm that most of the groups chose the correct option.

The next task was to select a list of 10 objects together, choosing from all the objects previously selected individually, based on physiological criteria and their personal experience.

The mean time for the students to reach a group decision and consensus was 20.5 ± 7.1 min. The group that reached consensus earliest did so in 9 min, while the group that took the longest took almost 38 min. Again, no significant relationship was found between the time taken to decide and the quality of the decision. Those who spent more time did not necessarily make better choices when compared to the list preconceived by the instructors. (Table 3). None of the teams managed to find the exact 10 items on the instructor’s list, but all of them matched at least four items.

**TABLE 3:** Agreement between group decisions and the optimal list of survival items. Comparison of the number of correct items selected by each group, percentage of agreement, average time to reach consensus, and time range.

Number of correct chosen objects	Number of groups who had chosen the correct objects	Groups’ percentage (%)	Average time spent choosing the objects (minutes± SD)	Range (minimum-maximum) (minutes)	95% CI (lower-upper)
4	1	4.2	19	—	—
5	9	37.5	19.9 ± 7.0	14.14–34.5	14.6–25.21
6	5	20.8	19.32 ± 4.55	14.5–26	13.67–24.97
7	6	25	17.73 ± 6.29	9–28.18	11.13–24.33
8	2	8.3	35.25 ± 3.18	33–37.5	6.66–63.83
9	1	4.2	18.9	—	—

### 3.4 Third phase: teamwork

Each team faced the challenge of answering 10 questions of varying types to test their knowledge and ability to apply it in the context of renal physiology. The questions were given one at a time, and teams had to answer each correctly before receiving the next. Impressively, all teams managed to complete the 10 questions within the stipulated time. This approach highlights the importance of the collaborative learning process and teamwork, emphasizing the progressive resolution of the challenges presented. By presenting the questions sequentially, it ensured that all team members worked together to find the correct answers, fostering a deeper understanding and cooperation.

### 3.5 Final score achieved

To evaluate how the different groups met the challenge of surviving in the desert, the instructors set up a point system based on the decisions made and the objects chosen.

The decision to stay and wait for help was valued with two positive points, while the decision to go for help was penalized with two negative points. Each item on the list ordered by the participants was assigned a different score by the instructors based on its prioritized order for survival in the desert (see Supplementary Table S1). Consequently, the final score depended on how the items were selected, as they did not all have the same value. Of the 29 items available to choose from, twelve of them had one positive point each, another twelve did not affect the score, and the remaining five subtracted one point. Therefore, the maximum score a group could obtain was 12 points (2 points for choosing to stay and 10 for selecting ten objects with positive points). When the activity was completed and the scores of each group were tallied, the average score for all groups was 7.88 ± 2.38.

### 3.6 Academic outcomes

Obviously, another aspect that we were most interested in knowing about was the influence that the dynamic might have had on the students’ learning process. To this end, we analyzed the grades received on the final evaluation test of the course and compared those students who participated in the group dynamics with those who did not.

**FIGURE 3:**
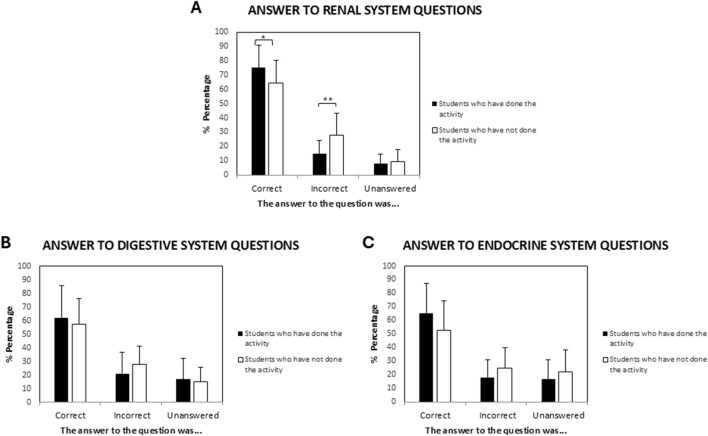
Academic performance comparison by content blocks between student cohorts. **(A)** Percentages of correct, incorrect, and blank answers for the renal physiology block. Students who participated in the group dynamics activity achieved a higher percentage of correct answers ( *p = 0.024) and a lower percentage of incorrect answers ( **p < 0.001) in their academic results. The effect sizes of these findings using Cohen`s conventions were medium and high for correct and incorrect answers, respectively. **(B)** Percentages of correct, incorrect, and blank answers for the digestive physiology block. **(C)** Corresponding results for the endocrine physiology block. No statistically significant differences were found in the percentage of digestive and endocrine questions answered.

The assessment consisted of 30 multiple-choice questions related to renal physiology. As shown in Figure 3A, the Mann-Whitney test was performed to compare the results of the students who participated in the activity with those who had not. Students who participated in the activity demonstrated a statistically significantly higher mean percentage of correct answers compared to non-participants (75.05% ± 15.96% vs. 64.07% ± 16.03; p = 0.024; d = 0.7, 95%CI [0.2 and 1.2]). In contrast, non-participants exhibited higher mean percentages of incorrect answers (27.83% ± 12.32% vs. 15.45% ± 9.95; p = 0.003; d = 1.1 95%CI [0.6 and 1.6]) and unanswered questions (9.5% ± 8.11% vs. 8.1% ± 6.62; p = 0,496). In all cases that are statistically significant, the p-values were adjusted by multiplying them by the number of comparisons that had been made. As shown in the data, the effect sizes of these findings using Cohen`s conventions were medium and high for correct and incorrect answers, respectively.

One potential explanation for these differences is lower engagement or commitment among non-participants. To explore this possibility, we analyzed performance in other thematic blocks of the course, specifically the digestive and endocrine systems, assessed using the same evaluation test.

In the digestive system block (Figure 3B), participants achieved a mean of 62.12% ± 23.5 correct answers, while non-participants achieved 57.27% ± 19 (p = 0.627). The mean percentages of incorrect answers were 21.07% ± 15.68% and 27.70% ± 13.67 for participants and non-participants, respectively (p = 0.111). Unanswered questions accounted for 16.81% ± 15.27 among participants and 15.03% ± 10.6 among non-participants (p = 0.286).

Similarly, in the endocrine block (Figure 3C), participants demonstrated a higher mean percentage of correct answers (65.39% ± 21.57) compared to non-participants (52.67% ± 21.79; p = 0.066). A comparable pattern was observed for incorrect answers (17.88% ± 13.27% vs. 25.03% ± 14.95; p = 0.123), whereas unanswered questions were more frequent among non-participants (22.20% ± 16.19) than participants (16.75% ± 13.92; p = 0.762).

These results suggest that non-participants tend to achieve lower scores across all thematic blocks. This trend is statistically significant in the renal system, however. This pattern may reflect an association between the renal system-focused group activity and higher performance, possibly related to better knowledge integration and application. Thus, the observed differences across blocks may reflect the activity’s effect on relatively higher performance in the renal system block rather than solely differences in general engagement.

This pattern may suggest that the renal system-focused group activity was associated with improved learning outcomes.

### 3.7 Students’ perceptions

Of the 140 students who participated in the group dynamics activity, 103 completed an anonymous survey about the activity. For ease of analysis, we categorized the 11 survey statements into three groups: those related to decision making (questions one through 7), those related to their perceptions of teamwork (questions eight through 11) and two final questions about their personal opinion about the group dynamics activity (questions 12 and 13) (See Supplementary Table S2).

#### 3.7.1 Students’ perceptions of decision-making and changes of opinion

Ease of decision-making: 90.3% of students found it easy to reach consensus on the priority order of items, while 9.7% faced challenges due to divergent opinions on the importance of items.

Changes in individual order: Only 14.6% kept their original order, while 85.4% changed their lists. Of those who changed, 90% did so because of persuasive arguments from classmates, while 10% did so to avoid prolonged disagreement. Of those who changed their order, 81.3% agreed that the arguments were scientifically supported, while 18.7% felt they were more intuitively based. None of the students believe that the arguments that were given were arbitrary and without any support to justify it.

Acceptance of changing ideas: 90.3% felt it was acceptable to change their ideas when others present alternative points of view, while 9.7% felt it was inappropriate. 95.1% found it easier to change their perspective when presented with reasoned, scientifically based arguments, while 4.9% hold a different point of view.

#### 3.7.2 Students’ perceptions of teamwork in group dynamics

Satisfaction with team assignment: 81.4% were satisfied with random group assignment, while 18.6% preferred to choose their teammates.

Enrichment from random assignment: 97.1% found working with randomly assigned teammates enriching.

Respect and comfort: 98% felt that team members were respectful of all opinions, and 98.1% felt comfortable working with randomly assigned classmates.

#### 3.7.3 Students’ perceptions of the overall evaluation of group dynamics

Preference for Future Repetition: 100% believe that the group dynamics should be repeated in future academic years for other students.

#### 3.7.4 Open-ended comments

The analysis of students’ responses to the group dynamics activity revealed several major themes, all reflecting a generally positive experience (Table 4).

**TABLE 4:** Representative student comments grouped by theme: The following are representative comments from students, taken from open-ended responses and grouped by theme.

Themes	Example quotes
Teamwork and Socialization (Collaborating with classmates, meeting new people, sharing different viewpoints …)	- “It has been a good opportunity to work with colleagues who I do not usually work with in class.” - “ It is a good way to socialize with the classmates and learn in a different way.” - “ It is important to reinforce the knowledge on the subject and to be able to know the different points of view of my classmates.”
Knowledge Reinforcement (Strengthening theoretical knowledge, reviewing concepts …)	- “I liked it, since we have to answer questions that can help us regarding the subject and reinforce teamwork.” - “It has helped me apply my knowledge to solve clinical cases.” - “If I had not done the activity, I would not have learned certain things well.”
Practical Application of Learning (Applying knowledge to clinical or practical cases …)	- “Very interesting and fun being able to apply what we have been taught in class to practical cases.” - “A wonderful way to learn and consolidate the study.” - “It is a very good way to better understand everything, to be able to work as a team in a different way.”
Dynamic and Fun Learning Environment (Enjoyable, motivating, dynamic …)	- “Very fun and a very dynamic way to learn with my teachers and classmates.” - “I found it a dynamic way to review class concepts and share ideas with other classmates.” - “It has surprised me a lot, it has been a lot of fun while we reinforced knowledge.”
Overall Positive Perception (High satisfaction, desire to repeat the activity …)	- “I would love to repeat it.” - “I found it a wonderful way to learn and strengthen the study of the subject.” - “I would love to do it again.”

##### 3.7.4.1 Teamwork and socialization

A significant number of students emphasized the benefits of working collaboratively. They valued the opportunity to interact with classmates they had not previously engaged with, fostering new connections and encouraging the exchange of diverse perspectives. Comments such as *“It has been a good opportunity to work with colleagues who I do not usually work with in class”* and *“It is important to reinforce the knowledge on the subject and to be able to know the different points of view of my classmates”* illustrate the perceived social and academic advantages of the teamwork approach.

##### 3.7.4.2 Knowledge reinforcement

Many students reported that the activity reinforced and consolidated their theoretical knowledge. The exercise allowed them to review key concepts and apply their learning in a meaningful way. Representative feedback included statements like *“I liked it, since we have to answer questions that can help us regarding the subject and reinforce teamwork”* and *“If I had not done the activity, I would not have learned certain things well”* indicating that the activity was instrumental in strengthening academic comprehension.

##### 3.7.4.3 Practical application of learning

Students highlighted the value of applying theoretical knowledge to practical cases. They appreciated the opportunity to engage with realistic clinical scenarios, which they found crucial for deepening understanding. For example, one student commented, “*Very interesting and fun being able to apply what we have been taught in class to practical cases*” demonstrating the effectiveness of the applied learning methodology.

##### 3.7.4.4 Dynamic and fun learning environment

The activity was widely perceived as dynamic, fun, and highly engaging. Students described the experience as more motivating and enjoyable than traditional lecture-based learning. Quotes such as *“Very fun and a very dynamic way to learn with my teachers and classmates”* and *“It has surprised me a lot, it has been a lot of fun while we reinforced knowledge”* support this observation.

##### 3.7.4.5 Overall positive perception

Finally, the general tone of the feedback was extremely positive. Many students expressed a strong desire to repeat similar activities, viewing them as both enjoyable and educational. Phrases like *“I would love to repeat it”* and *“I found it a wonderful way to learn and strengthen the study of the subject”* were recurrent among the responses.

## 4 Discussion

This study provides meaningful insights into how structured group dynamics influence both individual and collective learning processes in higher education. The sample, predominantly female (79.3%) with a mean age of 19, aligns with typical demographics in medical education, supporting the potential generalizability of findings to similar academic contexts.

The high participation rate (71.8%) indicates strong student engagement, suggesting that context-driven, problem-based scenarios—such as the survival activity used here—can serve as motivating and effective pedagogical strategies. Specifically, activities that integrate disciplinary content, like physiology, into applied challenges appear to stimulate interest and deepen understanding.

Analysis of the individual decision-making phase revealed that students spent significantly more time selecting survival items in the desert scenario than in the store-based task. This difference in time allocation likely reflects the greater cognitive demand of the desert task, which required integrating physiological concepts to make context-appropriate decisions. However, the lack of a significant association between time spent and decision quality suggests that extended deliberation alone does not guarantee better outcomes. This finding supports previous research indicating that decision accuracy in complex settings may depend more on conceptual understanding than on time investment (Kahneman, 2011; Shepherd et al., 2021).

Interestingly, no gender-based differences were observed in decision-making time, indicating that efficiency in processing and decision-making was similar across male and female participants in this academic setting.

In the group phase, students demonstrated a high level of collaborative effectiveness. Most groups (87.5%) arrived at the optimal decision - waiting for help - within a reasonable time frame. Notably, the time required to reach consensus did not predict decision quality, reaffirming that collaborative processes, rather than duration of discussion, are key to successful group outcomes.

Students’ perceptions of the group dynamics were overwhelmingly positive. A significant majority found consensus-building straightforward (90.3%) and were open to changing their initial perspectives when faced with compelling, evidence-based arguments. The willingness to revise one’s stance, reported by 85.4% of participants, underscores the role of peer influence and the value of scientific reasoning in collaborative settings. Importantly, 81.3% of students who changed their views believed the arguments presented were grounded in scientific knowledge, reflecting a critical and analytical approach to peer discourse.

Satisfaction with group composition was also high. Despite 18.6% expressing a preference for self-selected teams, 97.1% appreciated the learning experience with randomly assigned peers. Most participants felt respected and comfortable within their groups, suggesting that the activity promoted an inclusive, supportive environment conducive to both cognitive and interpersonal growth.

These subjective impressions were supported by objective academic results. Participation in the group dynamics activity was associated with significantly higher performance in the renal physiology block. Students who engaged in the activity achieved higher scores on the final assessment, showing a significantly greater proportion of correct answers and fewer incorrect or unanswered items (p ≤ 0.001). These differences were more pronounced than those seen in other content areas, such as digestive and endocrine physiology, where performance differences between participants and non-participants were not statistically significant. This suggests that collaborative activity may have had a positive influence on learning in the renal physiology block.

The differences obtained in the percentage of correct answers between the questions in the renal block, which were significantly higher, and the other two blocks could be associated with the fact that group dynamics were carried out in the renal physiology block. Nevertheless, causality cannot be definitively established due to the voluntary nature of participation. However, the convergence of perceptual, behavioural and performance data suggests the pedagogical value of structured group dynamics.

It is worth highlighting that both participants and non-participants were exposed to identical course content, taught by the same instructors, and under uniform teaching conditions. This methodological consistency minimizes the influence of potential confounding variables and strengthens confidence in the observed differences in academic performance to the group dynamics intervention rather than to instructional disparities.

This methodological consistency minimizes the influence of potential confounding variables and strengthens confidence in the observed association between participation in the group dynamics intervention and academic performance.

Moreover, students clearly valued the experience: all participants (100%) recommended repeating the activity in future cohorts. They emphasized the benefits of engaging with diverse perspectives, applying disciplinary knowledge in context, and developing soft skills such as communication, reasoning, and teamwork—competencies essential to professional development.

This study has several limitations that must be considered. The voluntary nature of student participation (71.8%) may introduce a self-selection bias, as those who participate tend to be more interested in the subject, which could affect academic performance. However, analyzing grades in other areas of the subject where no similar activity was carried out minimizes this bias, as students who participated in the group dynamic should also have performed better in sections on the digestive and endocrine systems.

Another important methodological limitation relates to study design and data analysis. Because the intervention was offered to the entire cohort and participation was voluntary, no *a priori* sample size estimation was conducted. All eligible students who wished to participate were included, which ensured full coverage of interested participants but limited statistical robustness. In addition, although the intervention was delivered in small teams, group identifiers were not recorded, so students were treated as independent observations in the analysis. This assumption may have led to underestimated standard errors. Future studies should therefore include power analyses and adopt hierarchical or cluster-adjusted statistical models to strengthen methodological rigor and analytical precision.

The study was conducted with a single cohort within a specific academic program, which may limit the generalizability of the results to other contexts. Furthermore, the absence of randomisation prevents definitive causal inference, and no baseline data on participants’ previous academic performance (e.g., prior grades in physiology, overall GPA) or class attendance were collected. Although all students were enrolled in the same academic year, received identical instruction, and were evaluated using the same assessment tools, the lack of baseline measures precludes adjustment for potential pre-existing differences between groups. Future research should address these limitations by including randomised controlled designs, systematic collection of baseline academic and demographic variables, and statistical adjustments for potential confounders.

Despite these constraints, the study provides valuable insights and lays the groundwork for future research. Subsequent studies could benefit from incorporating mixed-methods designs, involving a broader and more diverse student population, and exploring long-term effects on knowledge retention and skill development. Including variables such as student motivation, learning preferences, and group dynamics quality would also help clarify the mechanisms underlying the observed benefits. Overall, the results support further exploration of collaborative and active learning strategies to enrich academic experiences and foster meaningful learning in higher education.

Finally, this study provides promising associative evidence about the efficacy of group dynamics as a pedagogical tool, in line with previous research (Chau et al., 2025; Patel et al., 2025; Köse and Özcan, 2025). The ability to reach consensus, adapt ideas, and work collaboratively contributes to a richer educational experience. Effective implementation of group activities can develop transversal skills and meaningful learning in higher education (Widad and Abdellah, 2022). Our results suggest potential benefits of group dynamics activities for physiology learning, although causal relationships cannot be established from the current design. These findings align with previous literature supporting the use of active, student-centered methodologies in higher education (Hasibuan et al., 2025). Future research should focus on the need for randomised controlled trials with long-term follow-up to establish causality and retention effects.

To facilitate replication and adaptation of this educational strategy, we summarize key implementation details. The group dynamics activity was conducted in small groups of 5–6 students to promote equitable participation and effective teamwork. The intervention consisted of two structured sessions of 2 hours each, allowing sufficient time for decision-making, discussion, and feedback. Faculty members acted as facilitators, guiding the process, clarifying doubts, and ensuring that all participants contributed without directly influencing group decisions. The desert survival scenario can be easily adapted to other physiological systems by modifying the contextual challenges to match relevant mechanisms—for example, using hypoxia-based situations for respiratory physiology, nutrient absorption for digestive physiology, or hormonal regulation scenarios for endocrine physiology. These adaptations can maintain the same collaborative and decision-making framework while reinforcing system-specific learning objectives.

From a practical perspective, educators considering similar interventions should ensure careful planning of group composition, allocation of time, and facilitation resources, as these factors strongly influence the quality of group dynamics. Importantly, this approach can be adapted to other physiological systems beyond those explored in this study, provided that sufficient guidance, structured activities, and support materials are available. Such transferability may enhance students’ engagement and foster deeper understanding across multiple domains of physiology.

## Data Availability

The raw data supporting the conclusions of this article will be made available by the authors, without undue reservation.
