# Supplementary material for: Enhancing physiology learning through group dynamics: outcomes and perceptions of medical students
Source: Front Physiol. 2025 Oct 21;16:1662624. doi: 10.3389/fphys.2025.1662624 (PMC12584600; doi:10.3389/fphys.2025.1662624)
Supplement: Supplementary file 1 [file Table1.docx]

Supplementary Material

# Supplementary Figures and Tables

**Supplementary Table 1.** List of items available for selection in the store, along with instructors' scores and order of preference. Order of preference 1–10 represents the optimal set of items used for scoring; additional +1 items were considered beneficial but not part of the top 10.

| Item | Points | Order of preference |
| --- | --- | --- |
| Deodorant | 0 |  |
| Toothbrush and toothpaste | 0 |  |
| Hairbrush | 0 |  |
| Mirror | +1 | 6 |
| Wet wipes | 0 |  |
| Shower gel | 0 |  |
| Sandwich | -1 |  |
| Potato chips | -1 |  |
| Coffee | 0 |  |
| Candy bars | -1 |  |
| Nuts | -1 |  |
| Watermelon | +1 | 2 |
| Melon | +1 | 3 |
| Bananas | +1 | 5 |
| Tomatoes | +1 | 4 |
| Water | +1 | 1 |
| Juices | -1 |  |
| Soft drinks | 0 |  |
| Energy drink | 0 |  |
| Beer | 0 |  |
| Liquor | 0 |  |
| Raincoat | +1 | 10 |
| Tobacco | 0 |  |
| Lighter | +1 | 7 |
| Kitchenware | +1 |  |
| Napkins | +1 |  |
| Desert animal book | +1 | 8 |
| Desert Plants book | +1 | 9 |
| Magazines | 0 |  |
