# Supplementary material for: Enhancing physiology learning through group dynamics: outcomes and perceptions of medical students
Source: Front Physiol. 2025 Oct 21;16:1662624. doi: 10.3389/fphys.2025.1662624 (PMC12584600; doi:10.3389/fphys.2025.1662624)
Supplement: Supplementary file 2 [file Table2.docx]

Supplementary Material

**Supplementary Table 2.** Survey used with the students who carried out the group dynamics

1.- Was it easy to create a common list in your team?

*Yes/No*

2.- If you answered NO to the previous question, why do you think that was?

*Open question*

3.- Did you change the order of your individual list?

*Yes/No*

4.- If you answered YES to the previous question, it was because:

*To avoid arguing with my classmates / We ran out of time / They convinced me that it was a better option.*

5.- If I changed a question for my classmates, it was because:

*The arguments they gave me were based on scientific knowledge/The arguments they gave me were based on intuition.*

6.- Do you think it is bad to change the idea with which you come to work in a team?

*Yes/No*

7- Did you find it easier to accept another argument when the one you were given was based on scientific knowledge?

*Yes/No*

8- Would you have preferred to choose the group in which you would work?

*Yes/No*

9- Do you think it is enriching to work with a team that is not the one you would have chosen *a priori*?

*Yes/No*

10.- Do you think that the group respected the opinions of all the members of the team?

*Yes/No*

11- Did you feel comfortable and considered in the team?

*Yes/No*

12.- Do you recommend that we repeat this activity next year?

*Yes/No*

13.- What did you think about the kidney group dynamics? Write your sincere opinion.
